# The mechanisms of Pin1 as targets for cancer therapy

**DOI:** 10.3389/fimmu.2024.1482088

**Published:** 2024-11-18

**Authors:** Chuanfeng Liu, Lingying Dan, Quan Li, Ousman Bajinka, Xingxing Yuan

**Affiliations:** ^1^ Department of Pulmonary and Critical Care Medicine, Lishui Hospital of Traditional Chinese Medicine, Lishui, China; ^2^ Department of Endocrinology, Lishui Hospital of Traditional Chinese Medicine, Lishui, China; ^3^ School of Medicine and Allied Health Sciences, University of The Gambia, Banjul, Gambia; ^4^ Department of Gastroenterology, Heilongjiang Academy of Traditional Chinese Medicine, Harbin, China

**Keywords:** Pin1 inhibitor, peptidyl-prolyl isomerase, cis-trans structure, tumorigenesis, proline, anticancer therapy

## Abstract

Targeted therapy has considerable promise for the effective eradication of cancer at the primary tumor site prior to subsequent metastasis. Using this therapeutic approach, gaining an understanding of mechanistic cancer models is essential for facilitating the inhibition or suppression of tumor growth. Among different oncogenes and proteins, the protein interacting with never-in-mitosis kinase-1 (Pin1) is particularly important. The interaction between Pin1 and phosphorylated threonine-proline motifs results in significant alterations in protein structure and function. In this review, we provide a comprehensive summary of the processes involving Pin1 and its mechanisms in the context of cancer therapy. Pin1 enhances signaling pathways in a number of different human cancers and plays a pivotal role in the suppressive mechanisms relevant to cancer treatment. It is essential for the regulation of proline-directed phosphorylation and for modulating tumor suppressors. Inhibitors of Pin1, particularly naturally occurring substances, have been found to inhibit the carcinogenic activity of Pin1, and consequently this protein could represent an excellent candidate for novel cancer treatment strategies, offering a valuable therapeutic target in carcinogenesis and treatment resistance.

## Introduction

Protein interacting with never-in-mitosis kinase-1 (Pin1), a member of the peptidylprolyl *cis*-*trans* isomerase (PPIase) family, recognizes and catalyzes the isomerization of proline residues in proline-directed phosphorylation sequences, thereby altering the conformation of target proteins, which subsequently influences their function, stability, and interactions with other proteins (1). Pin1 isomerase, consisting of 163 amino acids and having a molecular weight of approximately 18 kDa, is encoded by the Pin1 gene located on chromosome 19p13.2 (2). It plays roles in a range of cellular processes, including cell cycle regulation, proliferation, apoptosis, metabolism, protein folding, transcriptional regulation, nuclear–cytoplasmic distribution, and protein degradation (3–5). In addition, Pin1 is strongly associated with the Notch, β-catenin, and PI3K/Akt/mTOR signaling pathways, as well as processes such as angiogenesis, differentiation, and epithelial-mesenchymal transition (6).

Pin1 is widely expressed in a range of cancers and is assumed to play a key role in tumorigenesis by modulating protein function and conformation. Cancer-driving signaling pathways are typically modulated by protein phosphorylation and dephosphorylation, and this distinctive property of Pin1 enables it to recognize and isomerize phosphorylated Ser/Thr-Pro moiety sequencing signals (7). Furthermore, the presence of Pin1 in cancer patients has been demonstrated to be associated with disease severity and poor clinical outcomes (8). Consequently, it is highly desirable to further elucidate the role of Pin1 in mediating the up-regulation of oncogenes, as well as the downregulation of tumor suppressors, which can effectively hinder malignant cell development.

Structurally, Pin1 comprises N- and C-terminal PPIase domains that play roles in mediating cellular processes and regulating multiple human cancers, including prostate cancer, breast cancer, oral squamous carcinoma, and nasopharyngeal carcinoma (NPC) (9, 10). Functionally, Pin1 is primarily involved in disrupting the regulation of the cell cycle. In addition to retinoblastoma protein (Rb), cell cycle regulatory proteins such as cyclin D1, cyclin E, Wee1, and Cdc25C, are also regulated by Pin1 (11). Furthermore, Pin1 has been shown to isomerize proteins associated with cell apoptosis, including Bcl-2 family members and epithelial mesenchymal transformation-related transcription factors, thereby enhancing cancer cell resistance to apoptotic signals and promoting their transformation to a mesenchymal phenotype, and thus enhancing their invasiveness and metastasis (12, 13). Additionally, Pin1 enhances drug resistance and promotes changes in the tumor microenvironment that are conducive to cancer cell survival (14).

Elevated levels of Pin1 have also been found to be associated with the promotion of chromosome stability (15). Consequently, therapeutic strategies targeting Pin1 could enhance genomic stability, inhibit cancer proliferation, and reduce metastasis. These features identify Pin1 as a prime candidate for targeted cancer therapy (16). Notably, in addition to its function as a proline isomerase, in recent years there has been a growing interest in the role of Pin1 in modulating cell death, functionality, and mutations in different cancers (17, 18). Given the importance of such mechanisms in the context of cancer research, in this mini-review, we present a comprehensive summary of the mechanisms of action involving Pin1. We also describe the different inhibitors of Pin1, including natural products and small molecules, and their potential applications in cancer therapy.

## Molecular mechanisms of Pin1

Pin1 has been established to coordinate the cell cycle in the process of cell division and participates in multiple signaling pathways (19). Recently, however, research has increasingly tended to focus on its carcinogenic implications within apoptosis signaling pathways and mechanisms of drug resistance. Pin1 has been found to confer significant resistance to DNA damage-induced apoptosis in cancer cells, potentially inhibiting a range of pro-apoptotic signals (20, 21). Following apoptotic stimulation, Bax and Bak induce programmed cell death by permeabilizing the outer mitochondrial membrane and releasing cytochrome *C* (22). Eosinophils in various of solid tumors exhibit functions that depend on the surrounding environment, and in response to cytokine stimulation, Pin1 attenuates apoptosis in human eosinophils by inhibiting the mitochondrial translocation of Bax (23). Furthermore, FADD and DAXX are key proteins that regulate the extrinsic cell death pathway. By downregulating DAXX via the ubiquitin-proteasome pathway, Pin1 has been found to inhibit DAXX-induced apoptosis in glioblastoma cells (24). In addition, by isomerizing FADD Ser194, Pin1 also promotes the sequestration of FADD in the cytoplasm of eosinophils, thereby inhibiting FAS-FADD-mediated apoptosis (25). Other apoptosis-regulating proteins, such as p53, promyelocytic leukemia (PML), and Rb, are also modulated by Pin1 (26, 27), and Pin1 also enhances p53-induced apoptosis and p53M-induced tumorigenic activities (28). Furthermore, there is evidence to indicate that Pin1 stimulates Bcl-2 dephosphorylation, which enhances its solidity and anti-apoptosis function. Surprisingly, a combination of Bcl-2 and Myc signaling may contribute to an increase in apoptosis abrogation and promote malignant development (29).

The combined inhibition of CDK1 and Pin1 using RO3306 and sulfopin has been shown to suppress orthotopic tumor growth (30). Moreover, Pin1 is an essential regulator in managing histone deacetylase 6 (HDAC6), influencing the motility of the cells in lung cancer (31). In this regard, Pin1 has been established to interact with two phosphorylation sites on HDAC6, namely pSer22 and pSer412, to facilitate HDAC6-mediated cell motility. Studies focusing on the potential role of Pin1 in cholangiocarcinoma (CCA) have also revealed that by regulating ANXA2 phosphorylation, elevated levels of Pin1 expression enhance CCA cell proliferation and migration (32), whereas in gastric cancer, upregulation of Pin1 induces CREB1-activated PIN1P1, which plays a vital role in tumor progression (33).

In addition to disrupting the balance between tumor suppressors and oncogenes, Pin1 has been identified as a key player in cancer therapeutic resistance, notably in breast, pancreatic, and hepatocellular carcinoma (HCC) (34–36), which could potentially be treated based on therapy using a combination of Pin1 inhibitors and chemotherapeutic agents (37). Pin1 also reduces myeloid cell leukaemia-1 (Mcl-1), which enhances chemo-resistance, and is thus positively correlated with poor survival in human breast cancer patients (38). A further resistance-induced mechanism associated with Pin1 involves the PARP inhibitor. Pin1 regulates the degradation of BRCA1 protein via double-stranded DNA breaks. Consequently, BRCAness (similar to BRCA1 or BRCA2 gene mutation signatures) in cancer cells has been established to be associated with the inactivation of Pin1, which is essential for PARP inhibitor treatment, as it sensitizes cells (39). Furthermore, Pin1 can interact with the N-terminal domain region of the androgen receptor in prostate cancer. Consequently, an inhibition of Pin1 expression can contribute to a significant reduction in androgen receptor transcriptional activities, and thus combined treatment with a Pin1 inhibitor and ralaniten leads to cell cycle arrest, thereby serving as an antitumor strategy against castration-resistant prostate cancer xenografts (40).

## Transcriptional and post-translational regulation of PIN1 in cancer

Significant increases in the expression of Pin1 mRNA expression have been detected in response to the oncogene-mediated activation of E2F1 transcriptional factors such as H-Ras, Her2, p38, and PI3K. The induction of Pin1 transcription via E2F1 appears to be associated with the presence of an E2F1 consensus sequence in the Pin1 promoter region (41). Pin1 transcriptional activity is similarly stimulated upon the interaction between Notch1 and the Pin1 promoter region (42), whereas transcriptional repressors have been demonstrated to inhibit Pin1 transcription by interacting with the Pin1 promoter (43). Reductions in Pin1 transcription have also been observed in response to NF-κB activation and the induction of p53 transcription via HEPN1, and BRCA1, a well-established tumor suppressor gene, has also been found to control the transcription of Pin1. By binding to other proteins, BRCA1 plays a vital role in DNA repair, and BRCA1 mutations frequently occur during cancer development, causing cells to accumulate DNA damage (44).

MicroRNAs (miRNAs), a category of diminutive non-coding RNAs with an approximate length of 22 nucleotides, mediate the regulatory control of gene expression by interacting with the 3′-untranslated regions (3′-UTR) of target mRNAs (45, 46). Among these miRNAs, miR-200c has been identified as a key modulator of breast cancer stem cell-like cells (BCSCs), which are closely implicated in cancer proliferation, metastasis, drug resistance, and recurrence. thereby suppressing Pin1-driven BCSC activities and breast tumorigenesis (47). Similarly, miR-200b has been found to have a significant influence on breast cancer metastasis by directly targeting the 3′-UTR of Pin1 mRNA, thereby regulating Pin1 expression at the translational level (48). Furthermore, the seed region of miR-296-5p has been demonstrated to interact directly with the 3′-UTR of Pin1 mRNA, thereby influencing the proliferation and anchorage-independent growth of prostate cancer cells (49), whereas by modulating Pin1 expression, miR-140-5p and miR-874-3p have been found to inhibit cell growth and colony formation and promote apoptosis in HCC (50, 51). Moreover, by regulating Pin1, miR-628-5p not only inhibits the proliferation and colony formation of gastric cancer cells but also influences cell migration and invasion (52), and miR-370 and miR-150-5p have been found to have a significant influence on the transcriptional levels of Pin1 in esophageal squamous cell carcinoma (ESCC) and human laryngeal epidermoid carcinoma cells (53, 54).

**Figure 1:**
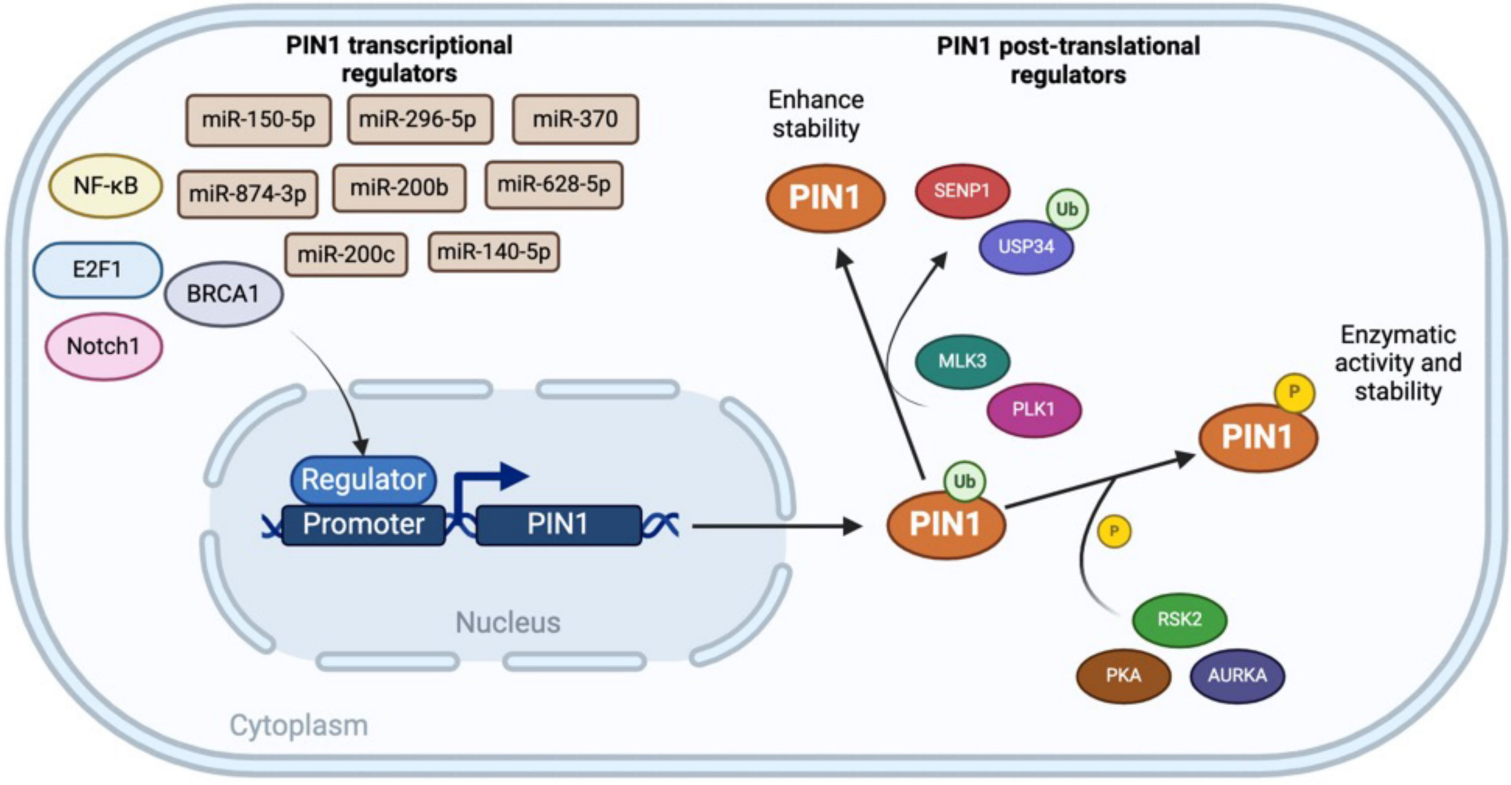
Transcriptional and Post-Translational Regulation of Pin1.

Under physiological conditions, protein function is often altered via post-translational modifications, and in this regard, the activity and functionality of the Pin1 protein have been shown to be post-translationally modified via oxidation, phosphorylation, SUMOylation, and ubiquitination (55). When subjected to oxidative stress, Pin1 is frequently oxidized at the Cys113 residue within the PPIase catalytic site, thereby reducing enzymatic activity (56). In addition, Ser16, Ser138, Ser65, and Ser115 residues in the Pin1 protein sequence have been identified as potential phosphorylation sites (57), and a reduction in phosphorylation at the Ser16 site within the N-terminal domain has been found to inhibit the binding of Pin1 to specific substrates. Plausible sources of this phosphorylation include ribosomal S6 kinase 2 (RSK2), protein kinase A (PKA), and aurora kinase A (AURKA) (58). Moreover, the phosphorylation of Pin1 at Ser138 via mixed lineage kinase 3 (MLK3) has been shown to enhance its nuclear localization and catalytic activity (59), whereas phosphorylation at the S65 site by polo-like kinase 1 (PLK1) prevents its poly-ubiquitylation and increases the levels of Pin1 protein (60). Furthermore, c-Jun N-terminal kinase (JNK) has been found to phosphorylate Pin1 at residue Ser115, thereby inhibiting monoubiquitination at Lys117, which prevents proteasomal degradation and facilitates the progression of intrahepatic cholangiocarcinoma (ICC) (61). SUMOylation of the N-terminal Lys6 and C-terminal Lys63 sites of Pin1 has been shown to regulate both oncogenic and enzymatic activities (62), whereas sentrin-specific peptidase 1 (SENP1) mediated deSUMOylation of Pin1 at Lys6 and Lys63 restores this protein’s substrate-binding and catalytic activities. Furthermore, in glioma stem cells (GSC), Pin1 undergoes deubiquitination and stabilization via the action of USP34, which is instrumental in facilitating glioblastoma (GBM) initiation, progression, and therapeutic resistance (63) (**Figure 1**).

## Mutation of PIN1 as a targeted cancer therapy

The cBioPortal is considered the primary resource for a thorough analysis of cancer genomics data (64). According to data from cBioPortal, a total of 11,000 cancer cases were submitted for genomic characterization in December 2013, along with 32,555 cases from 61 primary cancer sites (65). In total, over 41,924 distinct samples from different cancer patients with different cancer-related diseases have been identified (66). This source revealed the presence of 32 somatic mutations influencing 29 distinct locations in the Pin1 gene coding region. Among these, five mutations affect the WW domain, two are located within the flexible linker region, and 25 are found in the PPIase domain, of which 23 are missense mutations that do not alter Pin1 function, four are synonymous mutations, and four driver mutations. Overall functional analysis utilizing the sorting intolerant from tolerant (SIFT) algorithm identified 17 mutations as pathogenic and three as potentially harmful, based on structural data derived from the X-ray crystallography of Pin1 bound to a non-natural peptide inhibitor (48). The WW domain contains the Q33K and R36P mutations, whereas the PPIase domain includes all the other identified mutations. Among these, F134S, S154F, and H157Y interact with the enzyme’s substrates, whereas S71L, D112N, P133L, and T152M contribute to substrate communication via indirect pathways (67). The remaining mutations have no effects on substrate interactions, and thus instead may contribute to crosstalk with other proteins or influence the protein-folding dynamics of Pin1. One notable mutation is F139S, located at the interface of the PPIase domain, which by normalizing Pin1 function upon substrate binding, has been established to play an important role in interdomain interactions (68).

## The known inhibitors of PIN1

To date several inhibitors that target Pin1 with varying degrees of specificity and efficacy have been identified. Among these, Juglone, a natural plant compound, has been established to reduce Pin1 protein expression and inhibit the progression of prostate cancer by targeting Pin1 activity (69), whereas by binding non-covalently to the active site of Pin1, arsenic trioxide (ATO) inhibits and degrades Pin1, thereby suppressing its oncogenic function. Furthermore, by blocking Pin1, therapy based on a combination of ATO and all-trans retinoic acid (ATRA) has been proven effective against tumor-initiating cells, particularly in triple-negative breast cancer (70). Additionally, rhein exerts antitumor effects by interfering with the Pin1/c-Jun interaction, thus contributing to strategies for cancer prevention or therapy (71). Moreover, epigallocatechin 3 gallate (EGCG), a component of green tea, functions as a cancer chemo-preventive agent that can be applied to inhibit Pin1. X-ray crystallography has revealed that EGCG interacts with the N-terminal WW and C-terminal PPIase domains of Pin1, thereby impeding its function and attenuating the JNK signaling pathway (72). Moreover, cinobufacini injection (CI), an aqueous extract of *Cutis bufonis*, has been demonstrated to dose-dependently inhibit Pin1 enzyme activity, resulting in the impaired expression of Pin1 both *in vitro* and *in vivo*, and is used clinically in cancer therapy in China (73).

The inactivation of Pin1 protein function restores chemosensitivity, curtails the expansion of cancer stem cells and tumor growth, and may eventually block metastatic spread. Consequently, there is growing interest in designing highly selective inhibitors of this protein. One notable candidate in this regard is KPT-6566, a selective inhibitor that targets Pin1 by inducing reactive oxygen species (ROS) release and DNA damage. Moreover, on binding to the catalytic site of Pin1, a quinone-mimicking drug from KPT-6566, induces apoptosis in multiple types of cancers (74). By inhibiting Pin1, KPT-6566 has also been found to promote the cytotoxic effect of cisplatin (75). TME-001 [2-(3-chloro-4-fluorophenyl)-isothiazol-3-one], a competitive inhibitor of the catalytic structural domain of Pin1-PPPase identified in 2011, has been shown to inhibit cell proliferation in HeLa cells (76), and the pTide peptide has been demonstrated to enhance membrane permeability via fragments that bind to the octaarginine sequence of Pin1. This interaction subsequently inhibits intracellular Pin1 activity and the proliferation of different cancer cell lines, including HeLa and BT-474, whilst also elevating the levels of PML and SMRT (silencing mediator for retinoic acid and thyroid hormone receptors) (77). PiB, a further Pin1-specific inhibitor, is similarly characterized by an inhibitory mechanism that suppresses the growth of Pin1-containing cells and controls the expression of Nanog, a homeobox transcription factor involved in embryonic stem cell proliferation, renewal, and pluripotency. In addition, via suppressing the activity of Pin1, aetyl-11-keto-β-boswellic acid (AKBA) has been shown to inhibit prostate cancer by stabilizing cyclin D1 (78).

**Figure 2:**
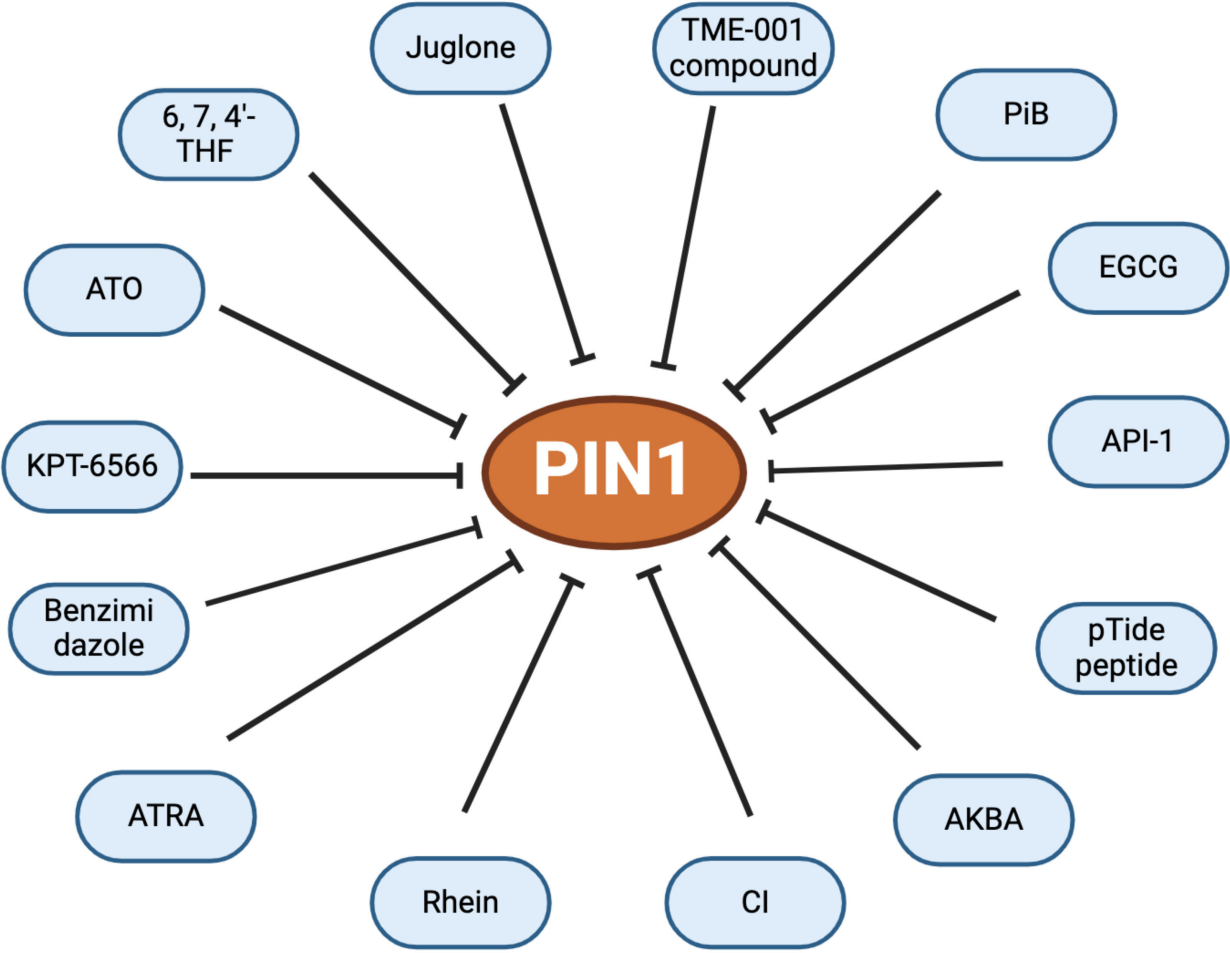
Known inhibitors of Pin1.

In addition to these specific inhibitors, structure-based drug design has successfully identified small molecules with a phosphate or phenyl imidazole core or carboxylate that specifically target the Pin1 protein. Based on of molecular docking analyses, 3D quantitative structure–activity relationships, and molecular dynamics simulations, it is observed that benzimidazole binds to the Pin1 protein (79), which could be attributed to electrostatic fields, hydrophobic interactions, and hydrogen bonding during the binding process between the inhibitor and target protein. Moreover, as key elements in the development of new anticancer drugs, benzimidazole derivatives can inhibit Pin1, thereby contributing to targeted treatment for prostate and breast cancer (80, 81). Among other similarly identified small molecules, computer virtual screening, based on an analysis of crystal structure, has revealed that API-1 binds to the PPI enzyme domain of Pin1. By regulating, miRNA biogenesis, API-1 has been shown to inhibit Pin1 and thereby serve as an antitumor agent for the treatment of HCC (82, 83). A further structural-based virtual screening study established that exposure of prostate cancer cells to a natural product-like inhibitor inhibited the interaction between Pin1 and the NF-κB p65 subunit, thereby resulting in a reduction of nuclear p65 (Thr 254) phosphorylation, thereby attenuating NF-κB activity and promoting apoptosis (84). Moreover, a virtual screening study identified the Pin1 protein as a target of 6, 7, and 4′-trihydroxyisoflavone (6, 7, 4′-THIF) (85). Notably, whereas the binding of 6, 7, 4′-THIF to the Pin1 protein was confirmed, this agent does not appear to interact with related proteins, such as FKBP and cyclophilin A (**Figure 2**).

## Future perspectives

In this review, we present a comprehensive overview of the roles played by Pin1 in the development of cancer from a holistic perspective by summarizing its pivotal involvement in biological functions, particularly within pro-apoptotic signaling pathways, and subsequently assessing its potential as a viable drug target. Additionally, the review has broad relevance across cancer types, as we assess the involvement of Pin1 in a range of cancer types and examine the combined effects of Pin1 inhibitors with other treatments such as PARP inhibitors and chemotherapeutic agents, thereby indicating their broad clinical applicability. Furthermore, our discussion regarding Pin1 as a therapeutic target is forward-looking, emphasizing its significance in cancer treatment and highlighting the potential of diverse Pin1 inhibitors, which provides valuable insights for advancing precision medicine.

Based on the information presented in this review, alternative strategies are recommended for developing Pin1 inhibitors, given its presumed role as a master cancer signaling regulator. Although the mechanisms underlying antitumor-associated inhibition of Pin1 have yet to be comprehensively established, the focus of research is increasingly shifting to an assessment of Pin1-targeting small-molecule compounds. These compounds have been established to show anticancer activities that are compatible with other targeted therapies based on Pin1 (86). Pin1 increases the stability of p53 via genotoxic interactions. Whereas p53 is repeatedly transformed in cancerous cells, Pin1 enhances the p53M-induced gain of new functionalities, leading to destructive malignancies (87, 88), and might also subvert PML, thereby promoting the growth of breast cancer cells. Furthermore, to stimulate E3 ligase KLHL20-mediated PML deprivation, which accelerates the progression of prostate cancer. Pin1 also stabilizes the oncogenic fusion protein PML-RARα, which shifts the location of wild-type PML from nuclear bodies to multiple micro-speckles, resulting in suppressed maturation in promyelocytic leukemia. In addition, the suppression of Pin1 using certain molecular drugs has been established to inhibit several tumor suppressor genes, including runt-related transcription factor 3 and variegation 3-9 homolog 1, which promote the activity of a number of oncogenic signaling pathways, whilst also contribution to increased levels of PML and SMRT proteins (89).

However, despite our emphasis on the promising therapeutic potential of Pin1 inhibitors, recent studies have highlighted possible side effects or toxicity issues associated with the use of such agents. Given the pivotal roles played by Pin1 in numerous cellular functions, inhibiting its activity could result in significant adverse side effects. Accordingly both the practical application of such inhibitors and their potential side effects require further study (90). For example, although efficient, the doxorubicin and ATRA combinatory therapy is linked to chemotherapy-induced side effects via Pin1 degradation (91). In addition, current drug development should focus on designing highly selective Pin1 inhibitor based on analyses of structure-specific binding sites and computer-aided drug design to optimize target activity. Furthermore, the cellular permeability of inhibitors can be enhanced by nanotechnology and effective drug delivery systems, such as encapsulating inhibitors within liposomes or polymer nanoparticles, to enhance their bioavailability.

## Conclusion

The types of cancers in which Pin1 is specifically implicated include prostate cancer, breast cancer, GBM, oral squamous cell carcinoma, NPC, gastric cancer, HCC, CCA, ICC, endometrial carcinoma, ESCC, acute myeloid leukemia, and pancreatic cancer. Pin1 plays essential roles in regulating Pro-directed phosphorylation and controlling tumor inhibitors. Modifying target proteins can influence protein function. As an inhibitor, the naturally occurring substances suppressing cancerous activities by interacting with Pin1. As a valuable therapeutic target in carcinogenesis and drug resistance, Pin1 can serve as an excellent candidate for the development new cancer treatment strategies. Additionally, it can influence immune surveillance via several mechanisms. However, the efficacy of using Pin1 as a target in cancer therapy is highly dependent on the cellular context.
